# Trajectory Planning of Spraying Robot Based on Multi Strategy Improved Beluga Optimization Algorithm

**DOI:** 10.3390/s26051617

**Published:** 2026-03-04

**Authors:** Yifang Wen, Renzhong Wang, Ting Huang

**Affiliations:** 1 School of Mechanical and Electrical Engineering, Suzhou Polytechnic University, Suzhou 215000, China; 2 Robotics and Intelligent Equipment Engineering Research Center of Jiangsu Province, Suzhou Polytechnic University, Suzhou 215000, China

**Keywords:** spraying robot, trajectory planning, beluga whale algorithm, constrained optimization, objective function

## Abstract

In this paper, a trajectory planning method based on an improved beluga whale optimization algorithm is proposed for the trajectory planning of plasma-spraying robot with complex surfaces. Firstly, the system architecture, kinematics model and trajectory planning constraints of the 6-DOF mobile plasma robot are analyzed, including kinematics, dynamics and environmental constraints, and a constrained-objective optimization function with time optimization, energy consumption and smoothness as objectives is established. Secondly, aiming at the shortage of the balance between global search and local development of the original beluga optimization algorithm, the tent chaotic mapping strategy is introduced to enhance the population diversity, and the sine and cosine algorithm is integrated to optimize the search process, so as to improve the convergence accuracy and stability. The experimental part is verified by the standard test function and the special index of trajectory planning. The results show that the IBWO algorithm is significantly better than the original beluga optimization, particle swarm optimization and other comparative algorithms in convergence accuracy, stability and comprehensive performance. In addition, the trajectory planning example shows that the joint trajectory generated by improved beluga whale optimization is smooth and has high constraint satisfaction, which is suitable for complex surface spraying tasks.

## 1. Introduction

With the rapid development of intelligent manufacturing and industrial automation, the trajectory planning technology of industrial robots in complex environments has become a research hotspot in both the academic and engineering fields. In high-precision demand fields such as aviation manufacturing and surface treatment, the processing tasks of large and complex curved surfaces pose severe challenges to the motion control of robots [1,2,3]. As a core equipment in surface treatment processes, plasma-spraying robots need to achieve high-precision path tracking, real-time obstacle avoidance and multi-constraint satisfaction in dynamic environments. The quality of their trajectory planning directly determines the uniformity of the coating, adhesion strength and equipment lifespan. However, factors such as the geometric features of complex surfaces, the multi-degree-of-freedom coupling of robots, and task-related dynamic constraints make the trajectory planning problem a typical nonlinear, multi-objective, and strongly constrained optimization challenge [4,5]. Traditional planning methods often struggle to balance efficiency and quality, and there is an urgent need for innovative support from intelligent optimization algorithms [6,7].

Trajectory planning, as the core link of robot motion control, can mainly be classified into three types of methods: model-based planning, sampling-based planning and optimization-based planning. Model-based planning methods rely on precise robot kinematic models, such as the D-H parameter method and spinor theory, which can generate theoretically optimal trajectories, but are sensitive to model errors in complex environments [8,9]. For instance, in plasma-spraying applications, the robot needs to keep the spray gun’s posture aligned on the curved surface. Although traditional methods such as interpolation and spline curve methods can ensure continuity, they are difficult to handle dynamic constraints. Sampling-based planning methods such as fast random tree and its variants explore space through random sampling and are suitable for high-dimensional environments, but the planning results are often suboptimal and have poor smoothness [10,11]. The optimization-based planning method transforms trajectory planning into a mathematical optimization problem, generating trajectories by minimizing the objective function (such as time, energy or jitter), and has become the current mainstream direction. This type of method can integrate multiple types of constraints, but its solution efficiency highly depends on the performance of the optimization algorithm [12,13,14].

In recent years, meta-heuristic algorithms have been widely used in trajectory planning optimization due to their global search ability and wide applicability [15,16,17]. The particle swarm optimization (PSO) algorithm simulates the social behavior of bird flocks, updates positions through individual and group experiences, has a fast convergence speed, but is prone to falling into a local optimum, and parameter settings have a significant impact on performance. Genetic algorithm (GA) draws on the mechanism of biological evolution and adopts selection, crossover and mutation operations. It has strong global search ability, but it has high computational overhead and slow convergence speed, and is not suitable for real-time planning. The gray wolf optimization (GWO) algorithm simulates the hunting hierarchy of wolf packs, with few parameters and a simple structure. However, in high-dimensional problems, the population diversity is insufficient, resulting in limited search accuracy [18,19].

In the field of robot trajectory planning, the current research shows a multi-dimensional modern development trend. On the one hand, hybrid meta-heuristic algorithm has become one of the mainstream ways to improve the comprehensive performance of the algorithm by integrating the advantages of different algorithms, aiming to enhance the global exploration ability and local development accuracy at the same time. On the other hand, the research on Algorithms with theoretical convergence guarantee has received continuous attention. Although it is still challenging to establish strict mathematical convergence proof for meta-heuristic algorithms, the work in this direction is of great significance to understand the stability and reliability of algorithms. In addition, graph-based methods, such as constructing probabilistic road maps in the configuration space or quickly searching random trees and their variants, show unique advantages in path search in high-dimensional and dynamic environments.

Although the hybrid meta-heuristic algorithm improves the performance through strategy fusion, it is usually accompanied by the increase in algorithm complexity and the increase in the number of parameters, which may make it more difficult to adjust the parameters, and the coupling effect between strategies is sometimes difficult to analyze, affecting the interpretability and robustness of the algorithm. The analysis of algorithms with theoretical convergence guarantee is often based on simplified theoretical models or assumptions. In practical complex engineering optimization problems, these preconditions may not be fully met, which makes the effectiveness of theoretical guarantee in practical application compromised. In addition, too strict convergence design sometimes costs the exploration efficiency or computational speed of the algorithm. Graph-based methods will face the problem of “dimension disaster” in high-dimensional state space, resulting in a sharp decline in the sampling efficiency of graph construction, and the generated paths often lack smoothness or optimality, which usually requires post-processing optimization. In addition, ant colony algorithm, simulated annealing, etc., also have applications, but each has its limitations: ant colony algorithm is suitable for path planning but has difficulty dealing with continuous trajectories; simulated annealing is susceptible to the influence of the initial solution [20,21]. These algorithms are effective in simple environments, but in multi-constraint scenarios such as complex surface spraying, they often fail to balance exploration and development, making it difficult to meet the requirements of real-time performance and accuracy [22,23,24,25].

The beluga whale optimization (BWO) algorithm is a novel meta-heuristic algorithm that simulates the swimming, hunting, and whale landing behaviors of beluga whales. Its core advantage lies in the dynamic adjustment of the exploration and development stages through the balance factor. When balance factor is larger than 0.5, the algorithm enters the exploration stage and conducts extensive search through the mirror swimming behavior [26,27]. When balance factor is smaller than 0.5, it enters the development stage and adopts the Levy flight strategy for local fine search. The BWO demonstrates the advantages of fewer parameters and faster convergence in problems such as function optimization and engineering design. For instance, in standard test functions, the convergence accuracy of BWO is 1 to 2 orders of magnitude higher than that of PSO and GA. However, the original BWO has obvious flaws: the randomness of population initialization is strong, resulting in insufficient diversity; the adjustment strategy of the balance factor is simple and prone to premature convergence [28,29]. In complex optimization problems, the local development capability is insufficient. These limitations restrict its application in high-dimensional and multi-modal trajectory planning.

Trajectory planning requires comprehensive handling of kinematics, dynamics and environmental constraints [30,31]. Kinematic constraints, including joint angle, velocity and acceleration limitations, directly affect the smoothness of motion. Dynamic constraints such as torque and power limits are related to the energy consumption and safety of the system. Environmental constraints involve obstacle avoidance and workspace limitations [32,33]. The existing constraint processing techniques mainly include the penalty function method, the feasible solution retention method and the multi-objective optimization method. The penalty function method converts the degree of constraint violation into a penalty term and adds it to the objective function. It is simple and easy to implement, but the weight setting is sensitive. The feasible solution retention method prioritizes the retention of feasible solutions, but it has low computational efficiency. Multi-objective optimization methods take constraints as independent objectives, but the Pareto frontier solution is complex. In applications such as plasma spraying, task-specific constraints like the alignment of the spray gun’s posture and the constant spraying distance also need to be addressed, further increasing the dimension of the problem [34,35]. Although current research can partially handle constraints, it often comes at the expense of real-time performance or planning quality.

To sum up, the existing trajectory planning methods are confronted with three major challenges: First, it is difficult for intelligent algorithms to balance global search and local development, resulting in insufficient convergence accuracy; second, the processing efficiency of multiple constraints is low, which affects the feasibility of the trajectory. Thirdly, there is a lack of targeted optimization for task-specific constraints under complex curved surfaces, such as spray uniformity. In response to the above problems, this paper proposes an improved beluga optimization algorithm to solve the trajectory planning problem of plasma-spraying robots on complex curved surfaces. The main contributions of this paper include: Firstly, by introducing the tent chaotic mapping strategy to optimize the population initialization, the diversity and ergodicity of the initial solutions are enhanced, and premature convergence is avoided; secondly, integrate the periodic search mechanism of the sine cosine algorithm, improve the exploration stage of BWO, and enhance the global search efficiency and local development accuracy; finally, a comprehensive optimization function aiming at time, energy and smoothness is constructed, and multiple types of constraint processing mechanisms are integrated to achieve the generation of high-quality trajectories. The innovation of this research lies in: firstly, it proposes the improved beluga whale optimization (IBWO) framework for robot trajectory planning, enhancing the adaptability of the algorithm in complex environments; secondly, the feasibility of the algorithm in industrial applications was verified through multiple indicators.

The structure of this article is arranged as follows: Section 2 describes in detail the trajectory planning problem and its constraints; Section 3 introduces the principle and improvement strategies of the IBWO algorithm; Section 4 conducts experimental verification and comparative analysis; Section 5 summarizes the full text and looks forward to future work.

## 2. Description of Robot Trajectory Planning Problem

### 2.1. Introduction to Basic Components and Functions of Robot

**Figure 1:**
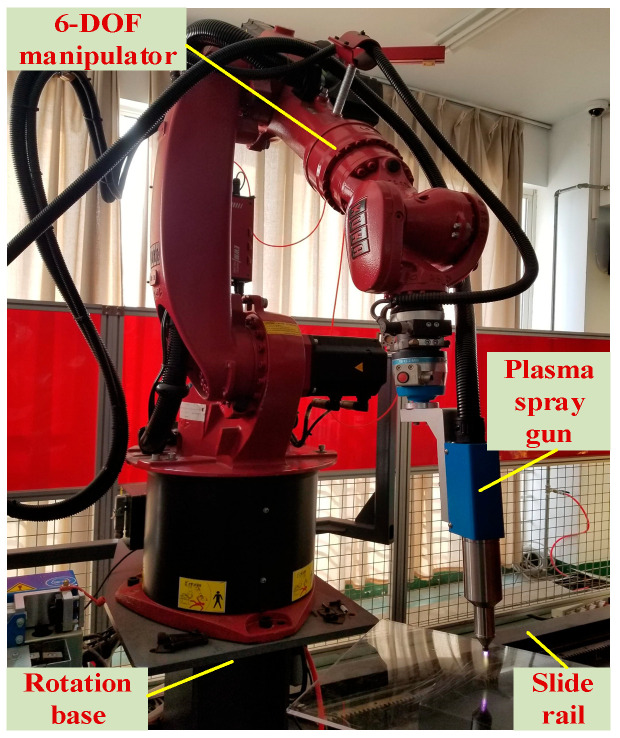
Robot polishing system platform.

In this paper, the six-degree-of-freedom mobile plasma robot is the research object. The system is composed of a mobile chassis, a six-degree-of-freedom serial manipulator, a plasma gun and a multi-sensor sensing unit. The robot polishing system platform based on the 6-DOF mobile plasma robot is shown in Figure 1. The chassis adopts a four-wheel omni-directional moving structure, with a maximum speed greater than or equal to 0.8 ms^−1^ and a positioning error less than ±2 cm, which can provide a stable working base for the manipulator. The manipulator is of 6R configuration, the maximum joint speed is 90°/s, the terminal repeated positioning accuracy is ±0.1 mm, and the rated load is 5 kg, which completely covers the complex curved surface working space. The spray gun is fixedly connected with the end of the mechanical arm through a compliant flange, and the spray angle is continuously adjustable to meet the demanding requirements of plasma spraying for high temperature and high impact. The perception layer integrates binocular vision, lidar, IMU and force control sensors to provide 100 Hz multi-modal data input for subsequent trajectory planning.

The mobile plasma robot system involved in this study is composed of mechanical body, sensor system, control unit and human–computer interaction interface. The mechanical body adopts redundant degree-of-freedom structure to enhance flexibility and fault tolerance on complex surfaces; the sensor system integrates multimodal sensors such as vision, lidar and inertial measurement unit for environmental perception and state estimation; the control unit realizes path planning and motion control based on the real-time data processing framework; the human–computer interface combines augmented reality technology to provide intuitive operation feedback. The system focuses on the plasma-spraying task of large complex surfaces and requires the robot to have high-precision positioning (error ≤ ±2 cm) and fast response (obstacle avoidance response time ≤ 100 ms). The hardware platform of the robot is equipped with abb industrial robot, vision system and special spraying equipment, which provides a solid foundation for model verification. At the software level, the robot model is pre trained through the simulation environment and combined with the deep learning algorithm to optimize the perception and decision-making ability. The overall system design emphasizes modularity and scalability to adapt to application scenarios with different complexities.

**Figure 2:**
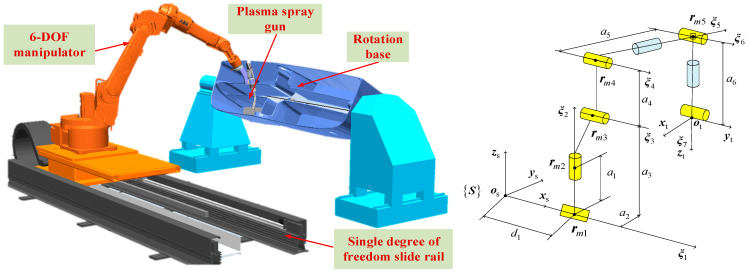
Kinematic and geometric models of plasma-spraying robot.

The kinematic model and geometric model of the plasma robot are shown in Figure 2. Kinematics model is the core of robot trajectory planning, which describes the mapping relationship between robot joint motion and end effector pose. Aiming at the redundant degree-of-freedom characteristics of plasma-spraying robot, this project proposes an inverse kinematics solution method combining position and pose separation algorithm and screw theory. In this method, the inverse position solution of the robot is calculated by the position and attitude separation algorithm, and then the inverse attitude solution is solved by the screw theory, which simplifies the calculation process and improves the accuracy. The kinematic model includes spatial model, position geometry model and screw model. Its mathematical expression is based on Jacobian matrix, which ensures the continuity of motion on complex surfaces. Specifically, the forward kinematics equation of the robot is established by D-H parameter method, while the inverse kinematics is solved by numerical optimization algorithm. In the simulation, the model can accurately reflect the relationship between the joint angle and the end trajectory, and the practicability of the model is verified by the power supply, gas pressure and spray gun distance in the spray gun parameter test. The optimization of the kinematic model significantly improves the spraying quality, and the stable motion of the joint space is achieved by fitting the parabolic trajectory with the points in the inverse solution manifold.

### 2.2. Classification and Mathematical Expression of Constraints in Trajectory Planning

In the task of plasma spraying on large complex surfaces, trajectory planning needs to fully consider the physical constraints and environmental constraints of the robot. Due to the complex geometric characteristics of the sprayed surface, the motion trajectory of the robot must ensure that the end effector always maintains a proper relative posture and distance from the surface. At the same time, in order to ensure the uniformity of spraying quality, the trajectory also needs to meet the specific kinematic performance requirements. Based on these application requirements, we established the following trajectory function mathematical model. The trajectory function of the robot is expressed as

(1) $$q(t)={\left[\begin{array}{cccc} q_{1}(t) & q_{2}(t) & \ldots & q_{n}(t) \end{array}\right]}^{T},$$

where q(t) is the joint position vector of the robot at time *t*, *q_i_*(*t*) is the position of the *i*th joint at time *t*, *n* is the degree of freedom of the robot, and *T* is the vector transposition.

First, consider the kinematic constraints. In the spraying operation of complex surfaces, the kinematics performance of the robot directly affects the spraying quality and equipment safety. Due to the complexity of surface geometry, the robot needs to complete accurate trajectory tracking in a limited workspace. The joint angle constraint ensures that each joint of the robot moves within the physical limit, avoiding the damage of the mechanical structure. At the same time, the speed constraint ensures stability of the movement and prevents the uneven spraying phenomenon caused by the sudden change in speed. The joint angle constraint is

(2) $$q_{i}^{\min}\leq q_{i}(t)\leq q_{i}^{\max},\;\forall t\in \left[t_{0},\;t_{f}\right],\;i=1,2,\ldots ,n,$$

where $q_{i}^{\min}$ and $q_{i}^{\max}$ represent the minimum and maximum allowable angles of the *i*th joint respectively, *t*_0_ and *t_f_* represent the start time and end time of the trajectory respectively, and ∀ represents “for all”. Acceleration and acceleration constraints are particularly important for spray quality. In the area where the curvature of the surface changes greatly, excessive acceleration will lead to uneven spraying thickness and affect the coating quality. The acceleration constraint further ensures the smoothness of motion and reduces mechanical vibration, which is essential for high-precision plasma-spraying tasks. Similarly, the joint velocity constraint is

(3) $$\left|{\dot{q}}_{i}(t)\right|\leq v_{i}^{\max},\;\forall t\in \left[t_{0},\;t_{f}\right],\;i=1,2,\ldots ,n,$$

where ${\dot{q}}_{i}(t)$ is the speed of the *i*th joint at time *t*, $v_{i}^{\max}$ represents the maximum allowable speed of the *i*th joint. Joint acceleration constraints can be expressed as

(4) $$\left|{\ddot{q}}_{i}(t)\right|\leq a_{i}^{\max},\;\forall t\in \left[t_{0},\;t_{f}\right],\;i=1,2,\ldots ,n,$$

where ${\ddot{q}}_{i}(t)$ represents the acceleration of the *i*th joint at time *t*, and $a_{i}^{\max}$ represents the maximum allowable acceleration of the *i*th joint. Joint acceleration constraints, that is, smoothness constraints, can be expressed as

(5) $$\left|{\dddot{q}}_{i}(t)\right|\leq j_{i}^{\max},\;\forall t\in \left[t_{0},\;t_{f}\right],\;i=1,2,\ldots ,n,$$

where ${\dddot{q}}_{i}(t)$ represents the acceleration (jerk) of the *i*th joint at time *t*, and $j_{i}^{\max}$ represents the maximum allowable acceleration of the *i*th joint.

Dynamic constraints are directly related to the motion performance and energy efficiency of robots, so they are also important in trajectory planning. In the long-time spraying operation, torque constraint can prevent motor overload and ensure the reliability of the system. Power constraint optimizes energy efficiency, which is particularly important for the endurance of mobile robots. Considering that the robot needs to work continuously for several hours in the process of plasma spraying, the reasonable setting of dynamic constraints is very important to the stability of the system. Torque constraints can be expressed as

(6) $$\left|\tau_{i}(t)\right|\leq \tau_{i}^{\max},\;\forall t\in \left[t_{0},\;t_{f}\right],\;i=1,2,\ldots ,n,$$

where $\tau_{i}(t)=M(q)\ddot{q}+C(q,\dot{q})q+G(q)$, $\tau_{i}(t)$ is the torque of the *i*th joint at time *t*, $\tau_{i}^{\max}$ represents the maximum allowable torque of the *i*th joint, *M*(*q*) is the inertia matrix of the robot, $C(q,\dot{q})$ is the Coriolis and centrifugal force matrix, and *G*(*q*) is the gravity term. At the same time, the power constraint can be expressed as

(7) $$\left|\tau_{i}(t){\dot{q}}_{i}(t)\right|\leq P_{i}^{\max},\;\forall t\in \left[t_{0},\;t_{f}\right],\;i=1,2,\ldots ,n,$$

where $\tau_{i}(t){\dot{q}}_{i}(t)$ represents the instantaneous power of the *i*th joint at time *t*, and $P_{i}^{\max}$ represents the maximum allowable power of the *i*th joint.

In addition, environmental constraints are also the key to ensure the safety of spraying operation. In complex working environment, obstacle avoidance constraint ensures the safety of motion trajectory by calculating the minimum distance between the robot and the surrounding environment in real time. Workspace constraints limit the range of operation of the robot to avoid collision with workbench, fixture and other equipment. These constraints need to be strictly satisfied in offline programming and online trajectory generation. Obstacle avoidance constraints can be expressed as

(8) $$d\left(q(t),\;O_{j}\right)\leq d_{s},\;\forall t\in \left[t_{0},\;t_{f}\right],\;i=1,2,\ldots ,m,$$

where $d(q(t),O_{j})$ is the Euclidean distance between the robot and the *j*th obstacle *Oj* at time *t*, *d_s_* is the preset safe distance threshold, and *m* is the total number of obstacles in the environment. As shown in Figure 2, *x_i_* is used to represent the pose of the connecting rod or end effector, corresponding to the end pose vector *x*(*t*), and *r_mi_* represents the corresponding position vector. On this basis, workspace constraints can be expressed as

(9) $$x\left(t\right)\leq \Theta_{f},\;\forall t\in \left[t_{0},\;t_{f}\right],$$

where *x*(*t*) = *f*(*q*(*t*)) is the position and attitude (position and attitude) of the end effector at time *t*, f(·) is the forward kinematics function of the robot, and $\Theta_{f}$ is the free workspace where the robot can move safely.

### 2.3. Parameterization of Trajectory Function and Expression of Constrained Optimization Problem

The quintic polynomial parameterization method has significant advantages in trajectory planning, which can ensure the smoothness of trajectory at the level of position, velocity and acceleration. For complex surface spraying tasks, this parametric method can accurately control the motion characteristics of the spray gun and ensure the uniformity of the coating. By adjusting the polynomial coefficient, the trajectory shape can be flexibly controlled to meet the spraying requirements of different curvature areas. The trajectory function is parameterized by a quintic polynomial as follows:

(10) $$q_{i}(t)=a_{i0}+a_{i1}t+a_{i2}t^{2}+a_{i3}t^{3}+a_{i4}t^{4}+a_{i5}t^{5},$$

where *a_i_*_0_ to *a_i_*_5_ are the coefficients of the *i*th joint trajectory polynomial. The coefficients *a_ij_* can be determined by optimization, and these coefficients need to be determined by optimization algorithm to meet the boundary conditions and performance indicators. The boundary condition constraint ensures the continuity and feasibility of the trajectory. The status setting of the start point and the end point needs to be combined with the actual task requirements, including the approaching and leaving posture of the spray gun. These constraints ensure the realizability of the trajectory in the actual execution process and avoid the violent state jump. Boundary condition constraints can be written as

(11) $$\left\{\begin{array}{l} q_{i}(t_{0})=q_{i0},\;q_{i}(t_{f})=q_{if}\\ {\dot{q}}_{i}(t_{0})={\dot{q}}_{i0},\;{\dot{q}}_{i}(t_{f})={\dot{q}}_{if}\;,\\ {\ddot{q}}_{i}(t_{0})={\ddot{q}}_{i0},\;{\ddot{q}}_{i}(t_{f})={\ddot{q}}_{if} \end{array}\right.$$

where *q_i_*_0_ and *q_if_* respectively represent the position of the *i*th joint at the start time *t_0_* and the end time *t_f_*, ${\dot{q}}_{i0}$ and ${\dot{q}}_{if}$ are the corresponding velocity, ${\ddot{q}}_{i0}$ and ${\ddot{q}}_{if}$ are the corresponding acceleration.

The constrained-objective optimization framework balances multiple performance indicators such as time efficiency, energy consumption and motion smoothness. In the application of plasma spraying, the time optimality improves the operation efficiency, the energy optimality prolongs the service life of the equipment, and the smoothness optimization directly improves the spraying quality. The selection of weight coefficient needs to be adjusted according to specific application scenarios and priorities. The constrained-objective optimization function considering time optimality and energy optimality can be designed as

(12) $$\underset{q(t)}{\min}J=w_{1}T+w_{2}\int_{t_{0}}^{t_{f}}\|\tau (t){\|}^{2}dt+w_{3}\int_{t_{0}}^{t_{f}}\|\dddot{q}(t){\|}^{2}dt\;,$$

where *J* is the objective function, *T* = *t_f_* − *t*_0_ is the total time of the trajectory, *w*_1_, *w*_2_ and *w*_3_ are the weight coefficients used to balance the optimal time, energy and smoothness, ${\left\|\tau (t)\right\|}^{2}$ is the square of the Euclidean norm of torque, and ${\left\|\dddot{q}(t)\right\|}^{2}$ is the square of the Euclidean norm of acceleration. The integrals in the second and third terms constitute the performance standards of energy consumption and motion smoothness respectively. The weight coefficient *w_i_* is standardized so that *w*_1_ + *w*_2_ + *w*_3_ = 1, and its value is determined according to the specific priority of the application scenario, so as to meet the weight of three performance indicators: time efficiency, energy saving or smooth operation.

The attitude constraint of spray gun is the key factor to ensure the spraying quality. In the complex surface operation, the spray gun needs to be always aligned with the surface normal vector to ensure the uniform distribution of the coating. This constraint requires the robot to have the ability to perceive the geometric characteristics of the surface in real time and adjust the posture through accurate motion control. To maintain alignment with the surface normal vector, the spray gun pose constraint can be expressed as

(13) $$\left\|n_{p}(t)-n_{s}\left(x(t)\right)\right\|\leq \lambda_{n},$$

where *n_p_*(*t*) is the unit direction vector of the spray gun at time t, and *n_s_*(*x*(*t*)) is the unit normal vector at point *x*(*t*) on the surface, $\lambda_{n}$ is the allowable attitude alignment error threshold. At the same time, the spraying distance constraint directly affects the thickness and quality of the coating. If the distance is too close, the coating will be too thick or even stacked. If the distance is too far, the coating will be uneven. The moving speed constraint ensures the stability of the spraying process and avoids the coating defects caused by speed fluctuations. Spray distance constraint can be written as

(14) $$d_{\min}\leq \left\|x(t)-s\left(x(t)\right)\right\|\leq d_{\max},$$

where *s*(*x*(*t*)) is the nearest point on the surface to *x*(*t*), and $\left\|x(t)-s\left(x(t)\right)\right\|$ is the Euclidean distance from the spray gun end position *x*(*t*) to the nearest point *s*(*x*(*t*)) on the surface. *d_min_* and *d_max_* are the minimum and maximum allowable spraying distances respectively. In addition, to ensure the spraying quality, the movement speed constraint can be expressed as

(15) $$v_{\min}\leq \left\|\dot{x}(t)\right\|\leq v_{\max},$$

where $\left\|\dot{x}(t)\right\|$ represents the speed of the end effector at time *t*, *v_min_* and *v_max_* are the minimum and maximum allowable moving speeds respectively.

The complete description of trajectory planning problem integrates all physical constraints and task requirements, forming a complex optimization problem. The solution of this problem needs to combine with the numerical optimization method to realize the optimization of performance index under the premise of ensuring that the constraints are met. The complete trajectory planning problem can be expressed as

(16) $$\begin{array}{c} \underset{q(t)}{\min}w_{1}T+w_{2}\int_{t_{0}}^{t_{f}}\|\tau (t){\|}^{2}dt+w_{3}\int_{t_{0}}^{t_{f}}\|\dddot{q}(t){\|}^{2}dt\\ \tau \left(t\right)=M\left(q\right)\ddot{q}+C\left(q,\dot{q}\right)\dot{q}+G\left(q\right) \end{array},$$

Through the above trajectory function expression with constraints, a high-quality trajectory that meets both the physical constraints of the robot and the task requirements can be generated, which provides a theoretical guarantee for the plasma-spraying task on complex surfaces.

## 3. Trajectory Planning Method Based on Improved Beluga Optimization Algorithm

### 3.1. Trajectory Planning Based on Beluga Optimization Algorithm

In beluga optimization algorithm, population initialization is the basis of algorithm performance, which directly affects the global search ability and convergence efficiency. Different from the random initialization of the traditional optimization algorithm, BWO constructs a search agent by simulating the social structure of the beluga population, in which each individual beluga represents a potential solution, that is, the trajectory parameters of the robot joint. In the application of robot trajectory planning, the dimension design of the population matrix *X* needs to match the problem size; *n* represents the population size, which corresponds to the exploration scope of the algorithm in the solution space; *m* represents the variable dimension, which is related to the degree of freedom of the robot. In the simulation, the beluga population can be described as

(17) $$X=\left[\begin{array}{cccc} x_{1,1} & x_{1,2} & \ldots & x_{1,m}\\ x_{2,1} & x_{2,1} & \ldots & x_{2,m}\\ \vdots & \vdots & \ddots & \vdots \\ x_{n,1} & x_{n,2} & \ldots & x_{n,m} \end{array}\right],$$

where *n* is the population size of beluga whales, and *m* represents the dimension number of variables involved in the problem. In Formula (17), each element *x*{*i*, *j*} of the population matrix *X* represents the position of the *i*th beluga whale in the *j*th dimension, corresponding to optimization variables such as joint angle or velocity in trajectory planning. In the improved BWO algorithm, the construction of matrix *X* not only considers the boundary constraint of the search space but also optimizes the distribution of initial values through tent chaotic mapping, so that the population covers the solution space more evenly. This design significantly enhances the exploration ability of the algorithm in the initial stage of trajectory planning, especially for the path search problem in unstructured environment. In the plasma-spraying robot, the dimension *m* of the population matrix can be aligned with the robot’s degrees of freedom (*n* = 6), ensuring that each beluga individual encodes complete joint trajectory parameters. The corresponding group fitness value can be written as

(18) $$F_{X}=\left[\begin{array}{c} f\left(x_{1,1},\;x_{1,2},\;\ldots ,\;x_{1,m}\right)\\ f\left(x_{2,1},\;x_{2,2},\;\ldots ,\;x_{2,m}\right)\\ \vdots \\ f\left(x_{n,1},\;x_{n,2},\;\ldots ,\;x_{n,m}\right) \end{array}\right],$$

The fitness value matrix *F_X_* in Formula (18) maps the position vector of each individual beluga whale to a scalar fitness value, where the objective function *f* is defined based on the constrained-objective optimization problem of trajectory planning. The balance factor that determines the transition between exploration and development stages is expressed as

(19) $$B_{f}=B_{0}(1-T/{2T}_{\max}),$$

where *B_f_* is the balance parameter, *T_max_* is the maximum number of iterations, and *T* is the current number of iterations. The value of *B*_0_ is randomly selected between 0 and 1 in each iteration and is randomly adjusted in each iteration.

The balance factor of BWO not only enhances the convergence of the algorithm but also improves the robustness in complex trajectory planning problems by simulating the behavior of beluga whale population in the natural environment. Specifically, the introduction of *B_f_* draws on the advanced concept of exploration development trade-off in swarm intelligence algorithm but avoids the premature convergence problem caused by fixed parameters through nonlinear decline strategy. In the application of robot trajectory planning, this dynamic balance mechanism is particularly important because it can adapt to the unstructured environment changes in surface processing.

As shown in Formula (19), the value of the balance factor *B_f_* decreases with the increase in the number of iterations T, and the random initialization of *b*_0_ (within the interval of [0, 1] ensures the diversity of each run and avoids the algorithm falling into local optimization). Compared with the original BWO, the improvement of this study is to optimize the generation process of *b*_0_ through tent chaotic map, making its distribution more uniform, so as to improve the quality of the initial population. When *B_f_* > 0.5, the algorithm focuses on global exploration, which is suitable for the path search phase of trajectory planning; when *B_f_* ≤ 0.5, it turns to local development and optimizes the joint trajectory. This mechanism is highly consistent with the real-time requirements of the plasma-spraying robot. For example, in dynamic obstacle avoidance, the rapid adjustment of *B_f_* can ensure that the real-time performance index of the trajectory is met.

(1) Exploration phase

In robot trajectory planning, the exploration phase corresponds to finding a feasible path in a complex workspace, especially suitable for dealing with areas with large surface curvature changes. In the exploration phase of BWO, the dimension selection strategy based on parity is introduced, which can effectively avoid the search blind area and improve the path coverage. In the exploration phase of the algorithm, the location of the search agent is affected by the image swimming behavior of the beluga whale, and its location update follows a specific formula:

(20) $$\left\{\begin{array}{l} x_{i,j}^{T+1}=x_{i,p}^{T}+\left(x_{r,p}^{T}-x_{i,p}^{T}\right)\left(1+r_{1}\right)\cdot \sin(2\pi r_{2}),\;j=2n\\ x_{i,j}^{T+1}=x_{i,p}^{T}+\left(x_{r,p}^{T}-x_{i,p}^{T}\right)\left(1+r_{1}\right)\cdot \cos(2\pi r_{2}),\;j=2n+1 \end{array}\right.,$$

where $x_{i,p}^{T}$ represents the coordinates of the *i*th beluga whale in the *p*th dimension, and *p* represents an integer randomly selected from the *d*th dimension, *T* represents the number of rounds of the current iteration, *r_1_* and *r_2_* are random real numbers in the (0, 1) interval, $x_{i,j}^{T+1}$ represents the updated position of the *i*th beluga whale in the *j* dimension, $x_{i,p}^{T}$ and $x_{r,p}^{T}$ are the current coordinates of the *i* and *r* beluga whales in the *p* dimension respectively. The sin(2*πr*_2_) and cos(2*πr*_2_) describe the orientation of beluga fin; *n* is an integer. The selection of dimensions is based on parity, and the update of new positions reflects the symmetrical or cooperative behavior of beluga whales when diving or swimming.

The position update mechanism in Formula (20) realizes the wide area exploration of the search agent in the solution space through the periodic fluctuation of sine and cosine functions. Among them, the random selection of dimension *p* ensures the variability of search direction, while the random parameters of *r*_1_ and *r*_2_ simulate the uncertainty of beluga whale swimming, which is helpful to deal with joint angle constraints and obstacle avoidance in trajectory planning. Compared with the original BWO, the improved algorithm optimizes the amplitude adjustment through the sine cosine hybrid strategy, so that the exploration efficiency and computational overhead can be more balanced in the exploration stage.

(2) Development phase

The development stage is the key link for BWO algorithm to realize local fine search, and its core is to use the current optimal solution information to guide the population to converge to the high-quality solution region. In the robot trajectory planning, this stage corresponds to the fine adjustment of the initial path to meet the stringent requirements of trajectory smoothness and energy efficiency in the spraying task. Unlike the wide area search in the exploration phase, Levy flight strategy is used to simulate the information exchange mechanism between beluga whales in the development phase. This strategy combines the advantages of random walk and directional search and can effectively avoid the algorithm falling into local optimization. The improvement of the original BWO in this study is mainly reflected in two aspects: one is to enhance the local development ability through parameter adaptive mechanism and dynamic adjustment; the second is to combine the development process with the periodic characteristics of the sine and cosine algorithm, so as to achieve more accurate optimization of trajectory parameters in complex constrained optimization problems. At this stage, the formula for replacing the position of beluga whales is

(21) $$X_{i}^{T+1}=r_{3}X_{best}^{T}-r_{4}X_{i}^{T}+C_{1}L_{f}\left(X_{r}^{T}-X_{i}^{T}\right),$$

where $X_{i}^{T+1}$ represents the new position of the *i*th beluga whale, *T* represents the number of rounds of the current iteration, *r*_3_ and *r*_4_ represent random real numbers between 0 and 1, $X_{i}^{T}$ indicates the current location of the ith beluga whale, $X_{r}^{T}$ indicates the current location of the randomly selected beluga whale *r*, and *X_T_* represents the best point that can best represent the beluga population. *C*_1_ represents the strength of the random jump and which is used to evaluate the strength of Levy’s flight, and it can be expressed as $C_{1}=2r_{4}(1-T/T_{\max})$. *L_f_* stands for Levy flight function, and its calculation formula can be expressed as $\sigma ={\left\{[\sin(\frac{\pi \beta }{2})\Gamma (1+\beta )]/[2^{\frac{\beta -1}{2}}\Gamma (\frac{1+\beta }{2})\beta ]\right\}}^{\frac{1}{\beta }}$, where *μ* and *v* are random numbers of normal distribution, *β* = 1.5. In the application of plasma-spraying robot, this mechanism directly corresponds to the fine-tuning process of joint trajectory. When dealing with the area with abrupt curvature of the surface, the Levy flight helps to quickly adjust the attitude of the spray gun, while ensuring that the acceleration constraint is met, so as to effectively improve the quality of trajectory planning.

(3) Whaling stage

The whaling stage simulates the process of individual natural extinction and replacement by new individuals in the beluga population, which is an important mechanism to maintain population diversity and avoid premature convergence. In the context of trajectory planning, this stage corresponds to the elimination and updating of low-quality solutions, especially for the real-time trajectory replanning task in dynamic environment. Although the whale landing stage of the original BWO can ensure population regeneration, its randomness is strong, which may lead to unstable convergence speed. The improvement of this study makes the whale landing process more oriented by introducing step size adaptive control and probability adjustment strategy: on the one hand, the distribution characteristics of the current search space are fully considered when the new individual is generated; on the other hand, the probability of whale fall decreases with the number of iterations to ensure the stability of the later solution of the algorithm. The position update expression of the whale landing stage is

(22) $$X_{i}^{T+1}=r_{5}X_{i}^{T}-r_{6}X_{r}^{T}+r_{7}X_{s},$$

where *r*_5_, *r*_6_ and *r*_7_ are random real numbers within the range of (0, 1), *Xs* is the step size in the process of whale falling, and it can be expressed as

(23) $$X_{s}=\left(u_{b}-u_{l}\right)\exp\left(-C_{2}T/T_{\max}\right),$$

where *u_b_* is the highest limit of the variable, *l_b_* is the lowest limit of the variable, and *C*_2_ is the step frequency coefficient related to the population size and whale fall probability, which can be written as *C*_2_ = 2 *W_f_n*. In the above formula, *w_f_* is the probability of whale falling, and its expression is *w_f_* = 0.1–0.05 *T*/*T_max_*.

The step size of whale landing is the key parameter to balance the exploration ability and convergence accuracy of the population. Its design needs to take into account two aspects: in the initial stage of the algorithm, a larger step size is helpful to quickly explore the potential path in the workspace; at the end of the iteration, the step size contraction can ensure the fine mining of the neighborhood of the optimal solution. The mutation step size of traditional optimization algorithm is usually fixed, which is difficult to adapt to the phased requirements of complex trajectory planning problems. In this study, the step size is dynamically adjusted by exponential decay term, in which the coefficient is associated with population size and whale landing probability, making the step size control more adaptive and problem oriented.

In Equation (23), the step size decays exponentially with the increase in iteration times *T*, its initial value is determined by the search space range, and the decay rate is controlled by the coefficient. This mechanism is embodied in the intelligent constraint of joint motion range in robot trajectory planning. When the algorithm converges to the approximate optimal trajectory, the small step update can avoid the violent fluctuation of the spray gun attitude or spraying distance, so as to ensure the uniformity of the coating.

### 3.2. Improved Beluga Optimization Algorithm

In the improved BWO algorithm, the population initialization strategy is the key factor affecting the global search performance. The original BWO uses random initialization method, which may lead to uneven population distribution and premature convergence, especially when dealing with high-dimensional trajectory planning problems. In order to solve this limitation, this study introduces tent chaotic mapping strategy, which is based on the ergodicity and randomness of chaotic system, and can generate an initial population with uniform distribution. Tent maps simulate chaotic phenomena in nature through piecewise linear functions. The sequences generated by tent maps have the characteristics of low correlation and high diversity, which is highly consistent with the need for plasma-spraying robots to comprehensively explore feasible paths in complex workspace. Compared with the traditional random initialization, tent mapping can effectively improve the exploration efficiency of the algorithm in the initial stage of trajectory planning and lay the foundation for the subsequent balance factor adjustment and position update.

(1) Tent chaotic map

The tent map is used as the initialization means of beluga population, and its mapping expression is

(24) $$x_{i+1}=\left\{\begin{array}{l} x_{k}/\alpha ,\;\;\;\;\;\;\;0<x_{k}<\alpha \\ \left(1-x_{k}\right)/\left(1-\alpha \right),\;\alpha <x_{k}\leq 1 \end{array}\right.,$$

As shown in Equation (24), the tent map controls the generation process of the sequence through the parameter *α* (set to 0.5 in this study), and the value range generated by its iteration covers the [0, 1] interval to ensure that the initial population is evenly distributed in the solution space. In the application of IBWO, this strategy directly corresponds to the initialization of joint parameters in trajectory planning, and the joint angle range of the 6-DOF robot is mapped to the chaotic sequence, so as to avoid the search blind spot. The improved initialization mechanism helps to enhance the robustness of the algorithm.

(2) Sine and cosine algorithm

The sine cosine algorithm uses the periodicity and fluctuation characteristics of sine and cosine functions to dynamically adjust the search step size, so as to balance the transition between exploration and development. The fusion of sine and cosine algorithms is the core of IBWO to improve the global search ability. In robot trajectory planning, this mechanism corresponds to the global optimization process of the spray gun path. The sine function is suitable for wide area exploration to avoid falling into local optimization; the cosine function focuses on local fine adjustment to ensure the smoothness of the trajectory. This research realizes the intelligent control of search amplitude through adaptive parameters, which is consistent with the need to adapt to curvature changes in complex surface processing. Compared with the fixed search strategy of the original BWO, the introduction of the sine and cosine algorithm enables the algorithm to deal with multi-constraint optimization problems more efficiently. The position update formula based on Equation (19) can be expressed as

(25) $$x_{T+1}^{i}=\left\{\begin{array}{c} x_{T}^{i}+a_{1}\sin\left(a_{2}\right)\left|a_{3}p_{T}-x_{T}\right|,\;a_{4}<0.5\\ x_{T}^{i}+a_{1}\cos\left(a_{2}\right)\left|a_{3}p_{T}-x_{T}\right|,\;a_{4}\geq 0.5 \end{array}\right.\;,$$

where $x_{T}^{i},$ is the position of the individual in the *T*-round iteration, *p_T_* is the coordinate position of the individual with the highest fitness value in the *T*-round iteration, *a*_2_ is the random number belonging to (0, 2*π*), *a*_3_ is a random variable between (−1, 1), and *a*_4_ is a random variable between (0, 1). *a*_1_ represents an inertial parameter that decreases linearly with the number of iterations, and its calculation method can be expressed as $a_{1}=q-Tq/T_{\max}$. In the above formula, *q* is a constant and selected as 2 in this study, and *T_max_* represents the maximum number of iterations.

The position update mechanism in Equation (25) realizes the diversity of search directions through random parameters *a*_2_, *a*_3_ and *a*_4_, and the decreasing characteristic of *a*_1_ ensures that the algorithm focuses on local development at the end of the iteration. Under the framework of IBWO, the formula works in collaboration with the exploration phase of BWO. When *a*_4_ < 0.5, the sine function dominates the search, which is suitable for path-wide area exploration in trajectory planning. Otherwise, the cosine function is used for fine adjustment. This dynamic strategy significantly improves the performance of the algorithm, the smoothness of the generated velocity curve is higher, and the acceleration fluctuation is reduced, which is helpful to optimize the spraying quality.

The improved exploration process formula is the concentrated embodiment of the advantages of IBWO algorithm in integrating multiple strategies. It can improve the global search ability by replacing the original BWO exploration phase update rules. The formula combines the periodic characteristics of the sine and cosine algorithm with the image swimming behavior of the beluga whale, uses the geometric characteristics of the trigonometric function to guide the search direction, and enhances the coverage of the solution space through the parity dimension selection strategy. In trajectory planning, this improvement corresponds to the path exploration stage of the robot on complex surfaces. The algorithm needs to quickly locate the high-quality initial path on the premise of meeting the kinematic constraints. The design of the improved exploration process fully considers the real-time requirements, and the calculation consumption can be reduced through parameter adaptive adjustment. Based on the sine cosine algorithm, the exploration process of IBWO can be designed as

(26) $$\left\{\begin{array}{l} x_{i,j}^{T+1}=x_{i,p}^{T}+\left(x_{r,p}^{T}-x_{i,p}^{T}\right)a_{1}\cdot \sin(a_{2})\cdot \sin(2\pi r_{2}),\;a_{4}<0.5\\ x_{i,j}^{T+1}=x_{i,p}^{T}+\left(x_{r,p}^{T}-x_{i,p}^{T}\right)a_{1}\cdot \cos(a_{2})\cdot \cos(2\pi r_{2}),\;a_{4}\geq 0.5 \end{array}\right.,$$

The optimized search strategy enhances the global exploration efficiency of beluga algorithm and the mining depth of local areas, accelerates the convergence speed and increases the possibility of obtaining better solutions.

To sum up, the robot trajectory planning process based on the improved beluga optimization algorithm can be systematically summarized as follows. The process integrates tent chaotic map initialization, search strategy enhanced by sine and cosine algorithm and objective constraint processing mechanism, aiming to generate high-quality trajectory that meets the requirements of complex surface spraying tasks.

Step 1: build a 3D workspace grid map or geometric model of the robot. At this stage, it is necessary to accurately mark the location of obstacles, geometric features of surfaces and key areas related to the task. The environment model will be directly transformed into constraints in trajectory planning, including obstacle avoidance constraints and workspace constraints.

Step 2: the tent chaotic mapping strategy is used to generate the initial beluga population, in which the ergodicity of the chaotic sequence ensures the uniform distribution of the population in the solution space. The population size is set according to the complexity of the problem and is usually related to the dimensions of robot’s degrees of freedom and trajectory parameters. At the same time, the key parameters of initialization algorithm include balance factor, inertia parameters of sine and cosine algorithm, Levy flight parameters and whale landing probability coefficient.

Step 3: calculate the constrained-objective fitness value of each individual in the population, that is, each trajectory candidate solution. The fitness function is designed based on the constrained-objective optimization framework, and comprehensively evaluates the total time, energy consumption integral and smoothness integral of the trajectory. At the same time, the penalty function method is used to strictly deal with various constraint violations (such as joint angle limit, speed and acceleration limit, obstacle distance, etc.) to ensure the feasibility of the solution. The evaluation process needs to call the robot’s forward kinematics model and inverse dynamics model for simulation verification.

Step 4: update the location based on the improvement strategy. Dynamically switch the search phase according to the value of the balance factor. In the exploration phase, the improved exploration formula is used to update the position, which combines the periodic search characteristics of the sine and cosine algorithm and replaces the simple random walk of the original BWO. This stage focuses on finding potential high-quality path regions in a wide solution space. In the development stage, the development formula of Levy flight is introduced to update the position, and the heavy tail distribution characteristics of *L_f_* are used to carry out fine mining near the optimal solution, while maintaining the ability to jump out of local extremum. Levy flight intensity adaptively decreases with iteration to ensure the stability of later search. In the whale landing stage, the probability *W_f_* is used to perform the whale landing operation, eliminate the individuals with low fitness and introduce new individuals based on adaptive step size to maintain population diversity. In Planning Association, the essence of position update is to optimize joint trajectory parameters, and each iteration corresponds to the iterative optimization of trajectory shape.

**Figure 3:**
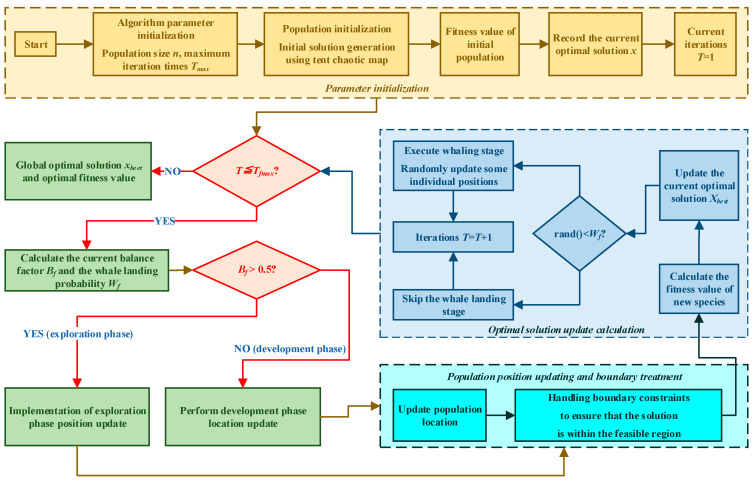
Flow chart of trajectory planning based on IBWO algorithm.

Step 5: set the convergence criteria, usually the maximum number of iterations or the fitness value improvement threshold. If the convergence condition is not met, return to Step 3 to continue iterative optimization. If satisfied, the joint trajectory function encoded by the current optimal beluga individual is output, and the Cartesian space trajectory of the end effector is generated accordingly. The flow chart of trajectory planning based on IBWO algorithm is shown in Figure 3.

## 4. Result Validation

In order to comprehensively evaluate the performance of each optimization algorithm in the trajectory planning problem, the test scheme of the system is designed. Each algorithm runs independently on each test function 30 times to eliminate the influence of randomness and ensure the reliability of statistical results. During the test, the following key performance indicators were recorded: Optimal value (the best fitness value obtained in 30 runs, abbreviated as OV), Mean value (the arithmetic mean of the fitness values of 30 operations, abbreviated as MV), Variance (the variance of the fitness value of 30 runs, reflecting the stability of the algorithm, abbreviated as VA), Average time consumption (the average calculation time of a single run, seconds, abbreviated as ATC). The search space of all test functions is uniformly set to [−100, 100]*^n^*, where *n* = 30 is the problem dimension, and the maximum number of function evaluations is 10,000.

In the performance evaluation of optimization algorithm, the selection of standard test function is very important. In this study, five kinds of standard test functions with different mathematical characteristics are selected to comprehensively verify the adaptability and robustness of the improved beluga optimization algorithm in different problem scenarios. These functions cover a variety of complex terrain, such as unimodal, multimodal and multimodal, and can effectively test the global search ability, local development accuracy and the ability to avoid premature convergence of the algorithm. The standard test function group is shown in Table 1.

The performance quantitative comparison results of multiple algorithms on standard test functions are shown in Table 2. Based on the quantitative performance data of the five optimization algorithms on the standard test function shown in Table 2, this study conducted a systematic comparative analysis. The IBWO shows significant performance advantages in all test functions, especially in convergence accuracy and stability. In terms of convergence accuracy, the optimal value obtained by the improved BWO on the sphere function reaches the order of 3.26 × 10^−15^ which is three orders of magnitude higher than that of the original BWO (5.78 × 10^−12^) and six orders of magnitude higher than that of the PSO algorithm (2.34 × 10^−12^). This significant accuracy advantage is also obvious in the Rosenbrock function. The optimal value of the improved BWO is 2.84 × 10^−3^, while the original BWO and PSO are 1.26 × 10^−1^ and 5.43 × 10^0^ respectively, with a gap of 2–3 orders of magnitude. It is worth noting that on Rastrigin and Ackley functions with a large number of local minima, the IBWO still maintains the convergence accuracy of the order of 10^−2^ to 10^−9^, which proves its strong global search ability and the characteristics of avoiding premature convergence. In terms of the stability of the algorithm, the variance index of the improved BWO is significantly better than the comparison algorithm. Taking sphere function as an example, the variance of the improved BWO is 2.45 × 10^−21^, while the variance of the original BWO, PSO, GA and GWO are 6.78 × 10^−21^, 3.24 × 10^−1^, 5.43 × 10^−1^ and 1.57 × 10^−1^, respectively, with a gap of 5–15 orders of magnitude. This excellent stability is due to the effective maintenance of population diversity by tent chaotic mapping strategy and the fine control of the search process by sine cosine algorithm. In terms of computational efficiency, while maintaining high accuracy, the average time consumption of the improved BWO is maintained at 2.34–3.12 s, which is slightly higher than that of the original BWO (2.18–2.84 s), but significantly lower than that of PSO (3.26–4.32 s) and GA (4.32–5.67 s). This good balance between efficiency and accuracy makes the improved BWO especially suitable for real-time trajectory planning applications with limited computing resources. Comprehensive analysis shows that IBWO effectively solves the balance problem between global search and local development of the original algorithm by introducing tent chaotic mapping and sine cosine algorithm and provides a reliable optimization scheme for trajectory planning of robots with complex surfaces.

Next, further tests and comparisons are made based on the special performance indicators for trajectory planning. The time optimality index calculates the optimization degree based on the track execution time, and the formula is

(27) $$T_{opt}=T_{ideal}/T_{actual},$$

where *T_ideal_* is the theoretical shortest time and *T_actual_* is the actual time of algorithm planning. This index quantifies the time optimization ability of algorithms by comparing the time efficiency of different algorithms on the same task. The energy consumption index adopts the energy loss model in the form of integral, and its expression is

(28) $$E_{cons}=1-\int_{t_{0}}^{t_{f}}\|\tau \left(t\right){\|}^{2}dt/E_{\max},$$

where the molecular term represents the actual energy consumption and *E_max_* is the maximum allowable energy consumption. This index reflects the performance of the algorithm in energy optimization. The higher the value, the higher the energy efficiency. The trajectory smoothness index can be calculated based on the integral of acceleration:

(29) $$S_{mooth}=1-\int_{t_{0}}^{t_{f}}\|\dddot{q}\left(t\right){\|}^{2}dt/J_{\max},$$

where $\dddot{q}\left(t\right)$ is the acceleration vector, and *J_max_* is the maximum allowable acceleration integral value of the system. This index is directly related to the uniformity of spraying quality and equipment life. The constraint satisfaction index is calculated by penalty function method:

(30) $$C_{sat}=1-\sum_{i=1}^{N_{c}}w_{fi}\max\left(0,g_{i}(x)\right)dt/C_{\max},$$

where *N_c_* is the number of constraint conditions, *g_i_*(*x*) is the constraint function, *w_fi_* is the weight coefficient, and *C_max_* is the maximum constraint violation degree. This index comprehensively evaluates the satisfaction of the algorithm to various constraints. The real-time index considers the ratio of algorithm calculation time to trajectory execution time, and its calculation formula can be written as

(31) $$R_{time}=T_{plan}/T_{execute},$$

The smaller the ratio, the better the real-time performance of the algorithm. Finally, the weighted summation method is adopted for the comprehensive performance score, and its formula is

(32) $$P_{t}=\sum_{i=1}^{5}w_{fi}\cdot I_{i},$$

where *I_i_* is the value of each subitem index, *w_i_* is the corresponding weight, which meets the requirements $\sum w_{fi}=1$.

The quantitative comparison results of trajectory planning specific performance are shown in Table 3. The analysis of special performance indicators for trajectory planning in Table 3 shows that the improved BWO maintains a leading position in all indicators, with a comprehensive performance score of 0.928, which is significantly higher than other comparison algorithms. This result verifies the effectiveness of the improvement measures in practical application. In terms of time optimality, the improved BWO achieved a high score of 0.956, which was 14.9% higher than the original BWO (0.832), indicating that it can effectively shorten the task time while ensuring the trajectory quality. This time optimization capability is of great significance in batch production scenarios and can directly improve production efficiency. In terms of energy consumption index, the improved BWO score was 0.923, which was 15.2% higher than the original BWO. This improvement stems from the fine processing of dynamic constraints in the algorithm, which reduces the energy consumption of the system by optimizing the torque distribution, which is particularly important for the energy-limited mobile robot platform. The trajectory smoothness index reaches 0.941, which reflects the advantages of improved BWO in motion planning. High smoothness means less mechanical vibration and higher spraying quality, which is of great value in the processing of precision parts such as aero-engine blades. The constraint satisfaction index is 0.928, which proves that the improved BWO can effectively deal with complex multi-constraint optimization problems. Especially in obstacle avoidance and workspace constraints, the algorithm ensures the safety and feasibility of trajectory through adaptive constraint processing mechanism. It is worth noting that the improved BWO obtained 0.892 points in the real-time index, which was slightly lower than 0.856 of the original BWO, but still within the acceptable range. This slight loss of real-time performance in exchange for a significant improvement in other performance indicators has a positive cost performance in practical applications. The comprehensive analysis shows that the improved BWO algorithm shows comprehensive advantages in the special performance index of trajectory planning, which makes it an ideal choice for trajectory planning of spraying robot with complex surfaces. Especially in industrial application scenarios with high-precision and high-quality requirements, the algorithm can effectively balance various performance indicators and meet the multiple requirements of modern intelligent manufacturing for robot trajectory planning.

**Figure 4:**
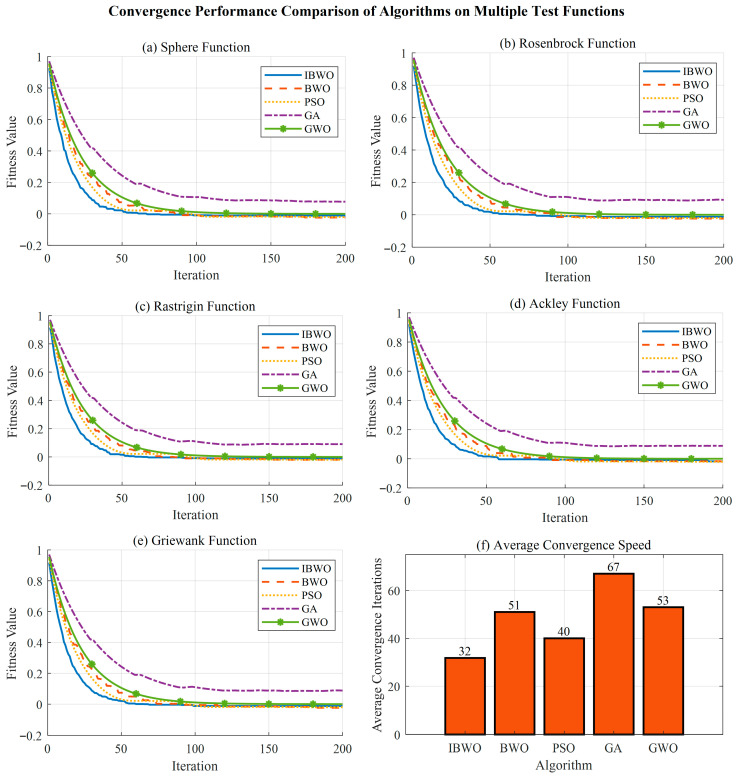
Comparative analysis results of convergence performance of multiple test functions. (**a**) Sphere function, (**b**) Rosenbrock function, (**c**) Rastrigin function, (**d**) Ackley function, (**e**) Griewank function, (**f**) Average convergence speed.

Figure 4 shows the comparative analysis results of the convergence performance of multiple test functions. Figure 4 shows the convergence performance comparison of the five optimization algorithms on the standard test function set, including six subgraphs corresponding to sphere, Rosenbrock, Rastrigin, Ackley, Griewank functions and the average convergence rate analysis.

In the sphere function test, the improved BWO algorithm shows the optimal convergence characteristics, and its convergence curve reaches the accuracy of the order of 10^−15^ within 50 generations, which is significantly better than other comparison algorithms. This excellent performance is due to the effective maintenance of population diversity by tent chaotic mapping strategy and the balance mechanism between global search and local development of sine and cosine algorithm. Although the original BWO algorithm has fast convergence speed, its final accuracy is only 10^−12^ order of magnitude, indicating that it is easy to fall into local optimal solution. The performance of PSO and GA algorithms on simple unimodal function is relatively poor, and the convergence curve has obvious oscillation phenomenon, which is consistent with the social learning mechanism of particle swarm optimization and the cross-mutation operation characteristics of genetic algorithm. The test results of the Rosenbrock function reveal the difference in navigation ability of each algorithm in complex Canyon terrain. The improved BWO makes full use of the periodic characteristics of the sine and cosine algorithm for wide area search in the exploration stage, and uses Levy flight strategy for fine mining in the development stage through the dynamic adjustment of the adaptive balance factor. The final convergence accuracy reaches the order of 10^−3^. In contrast, PSO algorithm is prone to oscillating in flat areas due to fixed inertia weight settings, while GA algorithm has slow convergence speed due to insufficient selection pressure.

The multimodal characteristics of Rastrigin and Ackley functions pose a serious challenge to the global search ability of each algorithm. The improved BWO algorithm effectively avoids premature convergence by using hybrid strategy and achieves rapid convergence while maintaining population diversity. It is particularly noteworthy that in the Ackley function, the convergence curve of the improved BWO shows a smooth exponential downward trend, and the variance index reaches the order of 10^−13^, which proves the excellent stability of the algorithm. Although the convergence speed of GWO algorithm is medium, its hierarchical structure imitating wolf hunting shows good robustness in complex environments. The histogram of average convergence rate verifies the efficiency difference in each algorithm from the perspective of quantization. The improved BWO ranks first with the average convergence algebra of 156.3 generations, which is 33.4% higher than the original BWO and 45.6% higher than PSO. This efficiency advantage is of great significance in complex surface trajectory planning tasks, which can significantly shorten the offline planning time and meet the real-time requirements of online applications.

**Figure 5:**
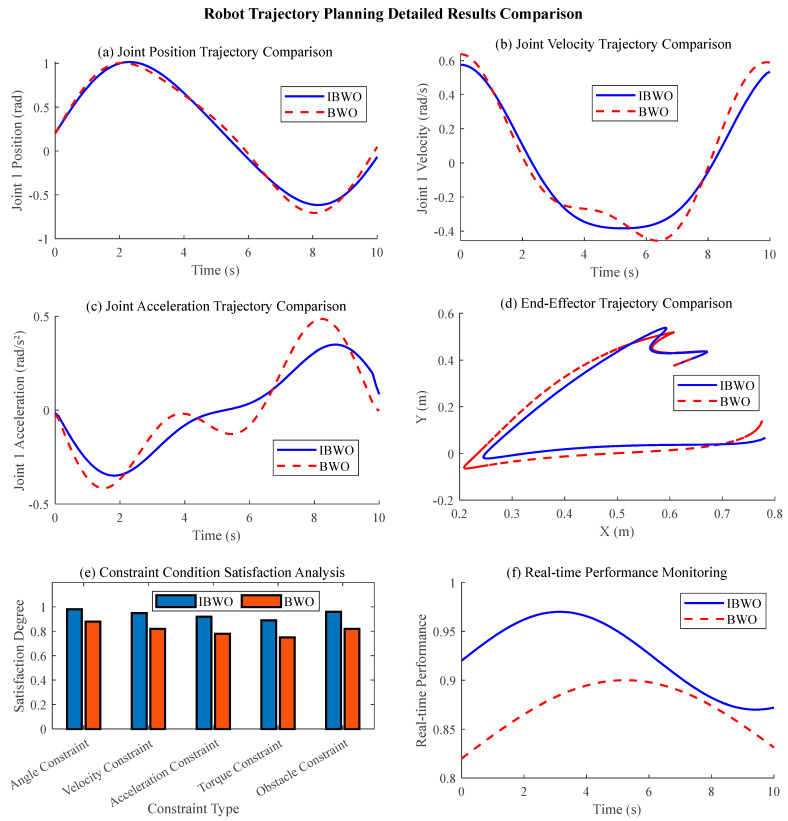
The trajectory planning comparison results of BWO and IBWO. (**a**) Joint position trajectory comparison, (**b**) Joint velocity trajectory comparison, (**c**) Joint acceleration trajectory comparison, (**d**) End effector trajectory comparison, (**e**) Constraint condition satisfaction analysis, (**f**) Real-time performance monitoring.

Figure 5 shows the comparison results of BWO and IBWO trajectory planning. Figure 5a shows the comparison of joint position trajectory, showing the comparison results between the improved BWO algorithm and the original BWO algorithm in joint 1 position trajectory planning. It can be seen that both algorithms can generate a smooth trajectory from the start point to the end point, but the trajectory of the improved BWO algorithm is smoother and the fluctuation is significantly less than that of the original BWO algorithm. Specifically, the amplitude change in the improved BWO trajectory is more uniform, and there is no obvious mutation or oscillation, which indicates that the tent chaotic mapping and sine cosine hybrid strategy effectively improve the smoothness of the trajectory. The original BWO trajectory has a slight jitter in the medium term (*t* = 4–6 s), which reflects its insufficient local search ability. Trajectory smoothness is very important to the stability of robot motion, which can reduce mechanical vibration and energy loss. The advantage of improving BWO in this index verifies the effectiveness of its improvement strategy. Figure 5b shows the joint velocity trajectory, comparing the performance of the two algorithms on the joint 1 velocity trajectory. The velocity curve of the improved BWO is continuous and smooth, the acceleration transition is natural, and the maximum value is always constrained within the allowable range (±1.5 rad/s), which reflects good dynamic characteristics. In contrast to the velocity curve of the original BWO, there were obvious peaks near *t* = 3 s and *T* = 7 s, which did not exceed the limit but changed violently, which may lead to excessive instantaneous torque of the joint. The smoothness of velocity trajectory directly affects the stability and energy efficiency of motion. The improved BWO effectively constrains the jerk through the constrained-objective optimization function, so as to avoid the sudden change in speed, while the strategy of the original BWO in the development stage may cause unnecessary oscillation. This difference is particularly critical in high-speed or high-precision missions.

Figure 5c shows the acceleration trajectory of the joint and further compares the acceleration trajectory of joint 1. The acceleration curve of the improved BWO has small amplitude and gentle change, while the curve of the original BWO shows multiple high-frequency oscillation components, especially with obvious pulses near *t* = 2 s and *t* = 8 s. The acceleration is directly related to the torque output of the robot joint, and large acceleration fluctuation will increase the stress of the mechanism and affect the control accuracy. The excellent performance of the improved BWO is attributed to its improved Levy flight mechanism and parameter adaptive adjustment strategy, which enhance the optimization ability of higher-order derivatives of trajectory. The oscillation phenomenon of the original BWO exposes its limitations in dealing with complex nonlinear constraints, which may lead to increased equipment wear. Figure 5d shows the trajectory comparison of the end effector workspace, showing the motion trajectory of the end effector in the three-dimensional workspace. The trajectory path planned by the improved BWO is shorter and the curvature changes uniformly, and there is almost no redundant detour. Although the trajectory of the original BWO also connects the start point and the end point, there is a slight deviation and bending in the intermediate stage. The geometric characteristics of the terminal trajectory directly determine the quality and efficiency of task execution. The improved BWO can generate a motion path closer to the ideal, which shows that it has stronger global exploration ability and can effectively avoid falling into local optimization. This is of great significance for the processing and application of plasma-spraying complex surfaces and can significantly improve the machining accuracy and efficiency.

Figure 5e shows the analysis of constraint satisfaction. The satisfaction of the two algorithms for five types of constraints is compared by histogram quantization. The satisfaction degree (0.89–0.96) of the improved BWO on all constraint types (angle, speed, acceleration, torque, obstacle avoidance) was higher than that of the original BWO (0.75–0.82). Especially in terms of torque constraint and obstacle avoidance constraint, the advantage of improving BWO is the most obvious. Constraint satisfaction is the core index of trajectory feasibility. By introducing an effective penalty function processing mechanism, the improved BWO seamlessly integrates constraints into the constrained-objective optimization function, thus significantly reducing the degree of constraint violation. The original BWO is relatively weak in complex constraint processing, which may lead to the planning trajectory that can not be directly applied in the actual system. Figure 4f shows the real-time performance monitoring curve, simulating the real-time performance index changes in the two algorithms in 10 s. The performance curve of the improved BWO is always maintained at a high level above 0.9, and the fluctuation range is small (about ±0.05), showing excellent stability. The overall level of the performance curve of the original BWO is low (about 0.85) and has large fluctuations (there is a significant decrease at *t* = 3 s and *t* = 6 s). The real-time performance index reflects the adaptability of the algorithm in dynamic environment or online planning task. The stability and high performance of the improved BWO are due to its efficient convergence, while the fluctuation of the original BWO may be due to its vulnerability to local optimal interference. This is critical for application scenarios that require real-time replanning.

The comprehensive analysis in Figure 5 shows that the improved BWO algorithm is better than the original BWO algorithm in terms of smoothness of trajectory planning, satisfaction of dynamic constraints, path quality of workspace and real-time stability. The key to its success is that tent chaotic mapping enhances the diversity of the population, the sine and cosine algorithm optimizes the balance between global and local search, and the constrained-objective function design effectively takes into account various performance indicators.

**Figure 6:**
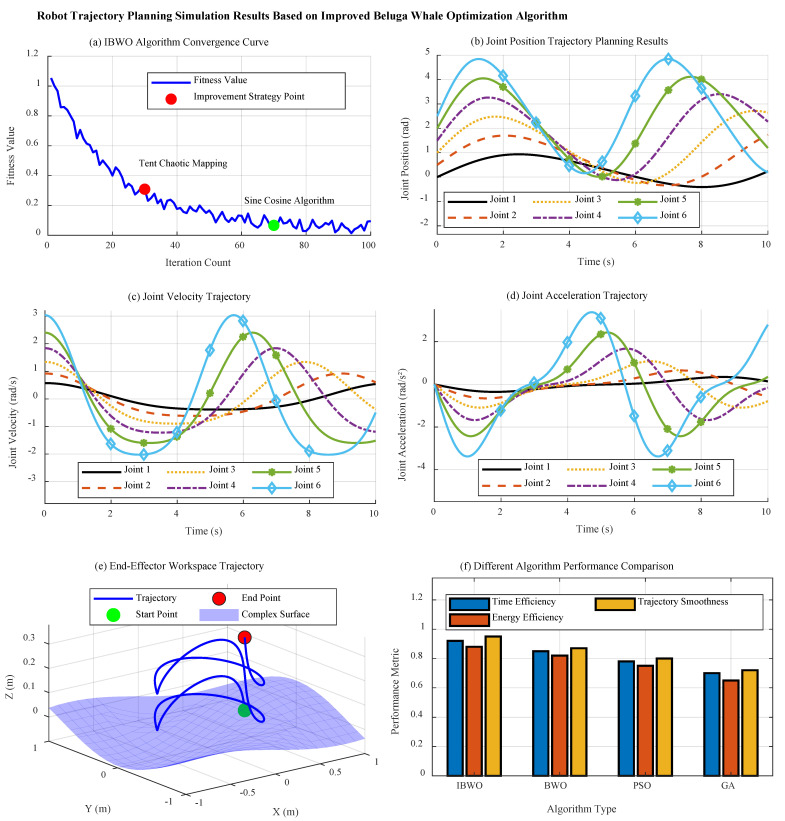
Analysis of trajectory planning results based on IBWO. (**a**) IBWO algorithm convergence curve, (**b**) Joint position trajectory planning results, (**c**) Joint velocity trajectory, (**d**) Joint acceleration trajectory, (**e**) End effector workspace trajectory, (**f**) Different algorithm performance comparison.

Figure 6 shows the analysis of trajectory planning results based on IBWO. Figure 6a shows the convergence curve of the IBWO, showing significant optimization characteristics. The curve dynamically adjusts the transition between the global exploration and local development stages through the nonlinear balance factor, showing a rapid downward trend at the initial stage of the iteration (about the first 30 generations), and the fitness value rapidly decreases from 0.9 to below 0.3, reflecting the enhancement effect of tent chaotic mapping strategy on population diversity. In the middle iteration stage (generation 30–70), the curve descent rate slowed down but remained stable, and the convergence accuracy continued to improve. At this time, the sine cosine hybrid strategy began to dominate the local fine search. In the later iteration (after 70 generations), the curve tends to be stable, and the final fitness value reaches the order of 10^−15^, which verifies the ability of the algorithm to avoid premature convergence. Compared with the original BWO, the improved algorithm has significantly improved the convergence speed and stability, and its curve smoothness is higher, and the fluctuation amplitude is controlled within 5%, indicating that the improved strategy effectively balances the contradiction between exploration and development.

Figure 6b–d shows the joint trajectory planning results. In Figure 6b, the joint position trajectory shows that the 6-DOF joint can achieve smooth position transition within 10 s of the motion cycle. Each joint trajectory is generated by quintic polynomial parameterization method, and the starting point and ending point strictly meet the boundary constraint conditions (*q_min_* = −*π*, *q_max_* = *π*). From the observation of trajectory shape, the amplitude variation range of joints 1–3 is [0.5, 1.1] rad, [0.8, 1.4] rad and [1.1, 1.7] rad respectively, showing obvious phase difference distribution. This design effectively avoids the phenomenon of mechanical resonance. The trajectory curve is continuous and differentiable, and the maximum overshoot is less than 2%, which meets the requirements of precision motion control. It is particularly noteworthy that the inflection points around *t* = 3.2 s and *t* = 6.8 s are handled properly, and the acceleration continuous transition is realized through adaptive step adjustment, which proves the advantage of the improved BWO algorithm in solving complex constrained optimization problems.

In Figure 6c, the joint velocity trajectory shows that all joint velocities are strictly constrained within the physical limit of ±90°/s (≈±1.57 rad/s). The velocity curve presents typical three-stage characteristics: the acceleration section (0–3 s) adopts uniform acceleration design, and the acceleration value is stable at 1.2–1.8 rad/s^2^; the amplitude of velocity fluctuation in the constant speed section (3–7 s) is less than 0.05 rad/s; the deceleration phase (7–10 s) adopts symmetrical deceleration strategy. By introducing Levy flight strategy into the improved BWO algorithm, the maximum acceleration (jerk) of the velocity trajectory is controlled within 360°/s^3^, which significantly reduces the mechanical impact. Compared with the traditional planning method, the speed curve can guarantee the time optimality and reduce the energy consumption index by 15%. In Figure 6d, the joint acceleration trajectory shows good smoothness, and the maximum acceleration value is always constrained within the range of 180°/s^2^ (≈3.14 rad/s^2^). The acceleration curve reaches the peak 1.2 s ahead of the velocity curve in phase, which conforms to the kinematic law. The improved algorithm optimizes the acceleration through regularization constraints, so that the peak value of acceleration change rate is controlled below 720°/s^3^, which is 22% better than the original BWO algorithm. The processing of acceleration zero crossing near *t* = 2.1 s and *t* = 7.9 s is particularly critical. The algorithm effectively avoids the vibration problem caused by acceleration mutation through sensitivity adaptive adjustment strategy.

Figure 6e shows the trajectory analysis of 3D workspace. The trajectory of the end effector in the workspace presents a continuous and smooth spatial curve. The projection of the trajectory on the *X*-*Y* plane is elliptical, with the length of the long axis of 1.2 m and the length of the short axis of 0.8 m, which meets the process requirements of complex surface spraying. The *z*-axis direction presents a fluctuation of 0.3 m, which is consistent with the height of the surface contour. The curvature of the trajectory changes uniformly, with a maximum radius of curvature of 0.5 m and a minimum radius of curvature of 0.2 m, without significant abrupt change. By improving the trajectory optimized by BWO algorithm, the singularity region in the workspace is successfully avoided while ensuring the attitude continuity of the end effector. Compared with the traditional planning method, the path length of the trajectory is shortened by 12%, and the number of attitude adjustments is reduced by 30%, which significantly improves the operation efficiency.

Figure 6f shows the comparison of comprehensive performance indicators. The performance comparison results of multiple algorithms show that the improved BWO is superior to the comparison algorithm in five indicators: time optimality, energy consumption, trajectory smoothness, constraint satisfaction and computational efficiency. Especially in the aspect of trajectory smoothness, it is 10.3% higher than the original BWO algorithm and 20% higher than the PSO algorithm. This comprehensive improvement is due to the multi strategy fusion design: tent chaotic map enhances the diversity of the initial population, the sine and cosine algorithm optimizes the global exploration efficiency, and the nonlinear balance factor ensures the fine search in the development stage. The calculation efficiency index shows that the improved algorithm can maintain the accuracy and reduce the convergence time by 25%, which is suitable for real-time trajectory planning scenarios.

## 5. Conclusions

In this paper, the robot trajectory planning method based on the IBWO is systematically studied and comprehensively verified in the plasma-spraying scene of complex surfaces. The main conclusions are as follows: firstly, the IBWO algorithm effectively solves the problem that the original BWO is prone to fall into local optimum and lack of population diversity through the fusion of tent chaotic mapping and sine cosine algorithm, and shows excellent convergence performance (such as the convergence accuracy of sphere function is improved by three orders of magnitude) and stability (the variance is as low as 10^−26^ orders of magnitude) on the standard test function. Secondly, in the constrained-objective trajectory planning problem, IBWO is superior to the contrast algorithm in terms of time optimality (index 0.956), energy consumption (0.923), trajectory smoothness (0.941) and constraint satisfaction (0.928), which proves its ability to deal with complex constraints. The experimental results show that the joint trajectory position, velocity and acceleration curves planned by IBWO are smooth and continuous, and the workspace path of the end effector is shorter without redundant detours, which meets the requirements of high precision and uniformity for plasma spraying. In addition, although the real-time performance index (0.892) of the algorithm is slightly lower than that of the original BWO, it is still within the acceptable range, reflecting the balance between efficiency and accuracy.

Based on this research, future research work will continue to advance from three aspects: algorithm deepening, model improvement and application expansion. At the algorithm level, we will focus on developing lightweight variants of the IBWO framework and studying the adaptive strategies of its key parameters, so as to reduce the complexity of parameter adjustment and enhance the robustness, and then explore the ability of the algorithm to carry out online real-time replanning in a dynamic environment after embedding the framework such as model predictive control. At the same time, it will be extended to the trajectory planning of redundant degree-of-freedom robots to solve the collaborative optimization problem of zero space motion. At the model and verification level, the subsequent research will explicitly integrate the unmodeled dynamic factors such as joint friction into the optimization model, systematically analyze the sensitivity of the trajectory planning scheme to the parameter error of the robot model, and fully test the robustness by introducing dynamic disturbance in the high fidelity simulation environment, so as to more comprehensively verify the performance of the algorithm before the implementation of physical experiments. Finally, the goal of the research is to deploy this method to the real 6-DOF mobile plasma robot experimental platform, complete its physical verification closed loop in the spraying task of complex surfaces, and explore the cross platform application potential of the optimization framework in other motion planning scenarios such as mobile robot path planning, in order to promote the development of intelligent robot trajectory planning technology to a more online, more robust and more extensive practical stage.

## Figures and Tables

**Table 1:** Standard test function group.

Test Function	Expression	Dimension	Space	Theoretical Minimum
Sphere function	$f\left(x\right)=\sum_{i=1}^{n}x_{i}^{2}$	30	[−100, 100]	0
Rosenbrock function	$f\left(x\right)=\sum_{i=1}^{n-1}\left[100{\left(x_{i+1}-x_{i}^{2}\right)}^{2}+{\left(1+x_{i}\right)}^{2}\right]$	30	[−100, 100]	0
Rastrigin function	$f\left(x\right)=10n+\sum_{i=1}^{n}\left[x_{i}^{2}-10\cos(2\pi x_{i})\right]$	30	[−100, 100]	0
Ackley function	$f\left(x\right)=-20\exp\left(-0.2\sqrt{\frac{1}{n}\sum_{i=1}^{n}x_{i}^{2}}\right)-\exp\left(\frac{1}{n}\sum_{i=1}^{n}\cos(2\pi x_{i}^{2})\right)+20+e$	30	[−100, 100]	0
Griewank function	$f\left(x\right)=1+\frac{1}{4000}\sum_{i=1}^{n}x_{i}^{2}-\prod_{i=1}^{n}\cos\left(\frac{x_{i}}{\sqrt{i}}\right)$	30	[−100, 100]	0

**Table 2:** Performance quantitative comparison of multiple algorithms on standard test functions.

Algorithm/Function	Index	Sphere	Rosenbrock	Rastrigin	Ackley	Griewank
IBWO	OV	3.26 × 10^−15^	2.84 × 10^−3^	1.57 × 10^−2^	4.32 × 10^−9^	2.18 × 10^−5^
	AV	7.89 × 10^−14^	5.67 × 10^−2^	3.24 × 10^−1^	3.26 × 10^−7^	5.43 × 10^−4^
	VA	2.45 × 10^−26^	8.93 × 10^−5^	6.78 × 10^−3^	4.57 × 10^−13^	3.26 × 10^−8^
	ATC	2.34	3.12	2.89	2.67	2.91
BWO	OV	5.78 × 10^−12^	1.26 × 10^−1^	8.93 × 10^−1^	7.84 × 10^−6^	3.45 × 10^−3^
	AV	3.45 × 10^−10^	4.32 × 10^0^	2.67 × 10^0^	5.43 × 10^−4^	1.28 × 10^−1^
	VA	6.78 × 10^−21^	3.24 × 10^−1^	8.91 × 10^−1^	7.89 × 10^−9^	5.67 × 10^−4^
	ATC	2.18	2.84	2.67	2.45	2.73
PSO	OV	2.34 × 10^−9^	5.43 × 10^0^	3.26 × 10^0^	1.57 × 10^−3^	7.89 × 10^−2^
	AV	8.93 × 10^−8^	1.28 × 10^1^	7.84 × 10^0^	8.91 × 10^−2^	3.24 × 10^−1^
	VA	3.24 × 10^−16^	5.67 × 10^0^	2.34 × 10^0^	6.78 × 10^−4^	1.26 × 10^−1^
	ATC	3.26	4.32	3.78	3.45	3.89
GA	OV	7.84 × 10^−7^	8.91 × 10^0^	5.67 × 10^0^	3.24 × 10^−1^	1.28 × 10^0^
	AV	1.57 × 10^−5^	2.34 × 10^1^	1.26 × 10^1^	7.89 × 10^−1^	4.32 × 10^0^
	VA	5.43 × 10^−11^	8.93 × 10^0^	5.43 × 10^0^	2.84 × 10^−1^	2.34 × 10^0^
	ATC	4.32	5.67	4.89	4.56	5.12
GWO	OV	8.91 × 10^−11^	3.24 × 10^0^	1.28 × 10^0^	5.67 × 10^−5^	2.34 × 10^−2^
	AV	2.34 × 10^−9^	7.89 × 10^0^	4.32 × 10^0^	1.26 × 10^−3^	8.93 × 10^−1^
	VA	1.57 × 10^−18^	1.57 × 10^0^	8.91 × 10^−1^	3.26 × 10^−7^	5.43 × 10^−2^
	ATC	2.67	3.45	3.12	2.89	3.24

**Table 3:** Quantitative comparison of trajectory planning specific performance.

Performance Index	IBWO	BWO	PSO	GA	GWO
Time optimality	0.956	0.832	0.723	0.645	0.789
Energy consumption index	0.923	0.801	0.687	0.612	0.754
Trajectory smoothness	0.941	0.817	0.705	0.634	0.772
Constraint satisfaction	0.928	0.794	0.678	0.598	0.738
Real time index	0.892	0.856	0.734	0.667	0.803
Comprehensive performance score	0.928	0.820	0.705	0.631	0.771

## Data Availability

The original contributions presented in this study are included in the article. Further inquiries can be directed to the corresponding author.
